# Diagnostic Modalities for Invasive Mould Infections among Hematopoietic Stem Cell Transplant and Solid Organ Recipients: Performance Characteristics and Practical Roles in the Clinic

**DOI:** 10.3390/jof1020252

**Published:** 2015-09-10

**Authors:** Ghady Haidar, Bonnie A. Falcione, M. Hong Nguyen

**Affiliations:** 1 Department of Medicine, University of Pittsburgh Medical Center, Scaife Hall, Suite 871, 3550 Terrace Street, Pittsburgh, PA 15261, USA; E-Mails: haidarg@upmc.edu (G.H.); FalcioneBA@upmc.edu (B.A.F.); 2 Department of Pharmacy and Therapeutics, University of Pittsburgh School of Pharmacy, 200 Lothrop St. 301, Pittsburgh, PA 15213, USA; 3 Department of Medicine, University of Pittsburgh, Scaife Hall, Suite 871, 3550 Terrace Street, Pittsburgh, PA 15261, USA

**Keywords:** invasive mould infections, aspergillosis, solid organ transplant, hematological malignancy, hematopoietic stem cell transplant, diagnostic biomarker, galactomannan, β-d-glucan, PCR

## Abstract

The morbidity and mortality of hematopoietic stem cell and solid organ transplant patients with invasive fungal infections (IFIs) remain high despite an increase in the number of effective antifungal agents. Early diagnosis leading to timely administration of antifungal therapy has been linked to better outcomes. Unfortunately, the diagnosis of IFIs remains challenging. The current gold standard for diagnosis is a combination of histopathology and culture, for which the sensitivity is <50%. Over the past two decades, a plethora of non-culture-based antigen and molecular assays have been developed and clinically validated. In this article, we will review the performance of the current commercially available non-cultural diagnostics and discuss their practical roles in the clinic.

## 1. Background

Invasive fungal infections (IFIs) are associated with substantial morbidity and mortality in transplant patients, and their incidence varies across different transplant populations. Prospective surveillance for IFIs by the Transplant-Associated Infection Surveillance Network (TRANSNET) identified an overall incidence of 3.4% (0.9%–13.2%) among hematopoietic stem cell transplant (HSCT) recipients [1] and a 12-month cumulative incidence of 3.1% (1.2%–6.1%) among solid organ transplant (SOT) recipients [2]. Even within transplant populations, the rates of IFIs differ. For example, among HSCT recipients, IFIs are more common among those receiving allogeneic transplants, particularly if HLA-mismatched or unrelated donors are used, those with graft-*versus*-host disease requiring high-dose corticosteroids and/or other immunosuppressive agents, and those with cytomegalovirus disease. Similarly, among SOT recipients, IFIs are more common in multivisceral and lung transplant recipients than other organs. *Candida* and *Aspergillus* are the most common fungal pathogens. However, with the widespread use of azole prophylaxis, the incidence of invasive candidiasis (IC) has substantially decreased, yielding ground to *Aspergillus* and other moulds such as *Fusarium*, *Scedosporidium*, and members of the order *Mucorales* (mucormycosis).

Early diagnosis leading to timely and optimal antifungal therapy improves outcomes [3]. Unfortunately, the diagnosis of IFIs continues to challenge clinicians. Traditional microscopy, histopathology and culture lead to IFI diagnosis in less than half of the cases. To date, there is not one specific test that can accurately diagnose IFIs, and we must instead rely on a combination of modalities, including clinical signs and symptoms in high-risk patients, radiographic findings, cytology, histopathology and culture of the affected site, as well as serological detection of antigens and other molecular methods. Over the past two decades, a plethora of non-culture diagnostics for IFIs have been developed and clinically validated. In this chapter, we will review the current commercially available diagnostic modalities for detecting IFIs. We will focus on *Aspergillus* and other common moulds, as the incidence of IC over the past decade has substantially decreased.

## 2. Conventional Assays

The gold standard for diagnosing of invasive mould infection (IMI) is culture of the affected site and the finding of tissue invasion by fungal pathogens on microscopy. In general, these methods are insensitive, but if positive, provide a firm diagnosis of IMI.

Direct microscopy of biological samples might allow for the rapid identification of infecting moulds. It is more sensitive than culture, since certain filamentous fungi, especially those belonging to the order *Mucorales*, might be damaged during the tissue homogenization step, or are difficult to grow *in vitro*. Microscopy, when enhanced with a fluorescent optical brightener (such as calcofluor white, brancofluor or FungiFluor) or stained with periodic-acid Schiff or Gomori methenamine silver, improves the detection of fungal pathogens. Fontana-Masson stain detects melanin, thus facilitating the diagnosis of phaeohyphomycosis. The major limitation of microscopy is its low sensitivity (<50%). In addition, unlike culture, microscopy cannot differentiate between members of the hyaline hyphomycetes (*Aspergillus*, *Fusarium*, *Scedosporidium*, *Paecilomyces*, *Acremonium*, *Phialophora*, *etc*) [4,5,6], and is likely to miss polyfungal co-infections. In addition, in some cases, such as with prior anti-mould antifungal therapy, the morphological features of *Rhizopus* may be atypical, thus reducing the ability to differentiate this genus from other mould species in histopathologic specimens [7]. Therefore, although not sensitive, culture should be routinely requested from appropriate specimens, since it will provide fungal identification and enable *in vitro* antifungal susceptibility testing.

Immunohistochemistry (IHC) and *in situ* hybridization (ISH) have been used to identify fungal pathogens to the genus level. IHC can be performed on fresh, frozen or paraffin embedded tissues using monoclonal or polyclonal antibodies raised against specific pathogen surface antigens. IHC has been applied to *Aspergillus* [5,8] and *Mucorales* [9,10]. ISH is more sensitive than IHC and can speciate fungal pathogens among the hyalohyphomycoses, phaeohyphomycosis and mucormycosis groups using species-specific probes [5,11,12,13,14,15,16]. The published upward sensitivity and specificity of ISH in diagnosing *Aspergillus* in tissues are 88% and 100%, respectively [13,14]. The advantages of IHC and ISH are their rapid turn-around time compared with culture and their ability to identify etiologic agents in the setting of negative cultures. Disadvantages of the tests are false positive (for example, antibody against *Aspergillus* might cross-react with *Fusarium*) [13] and false negative tests in necrotic tissues. IHC and ISH are not yet FDA approved, and their use requires laboratory internal validation.

## 3. Radiology

IFIs should be considered in transplant recipients with persistent fever and/or new pulmonary symptoms during substantial immunosuppression. Invasive moulds typically cause pulmonary infections but can also involve the sinuses and other extra-pulmonary sites. The threshold for performing radiography should be very low in these patients, especially when surveillance biomarkers are positive. Plain chest radiographs have limited diagnostic utility, and high-resolution computed tomography (HRCT) has become the imaging modality of choice for early diagnosis of IMIs. In patients with hematological malignancies (HM) and febrile neutropenia, systematic HRCT aids in the early diagnosis of pulmonary IMI, and early empiric anti-mould antifungal therapy in this setting has led to improved survival [3,17,18,19].

While numerous radiographic findings have been described on chest HRCT (dense masses, nodules with or without cavitation, focal, patchy or diffuse ground glass opacities, segmental or diffuse consolidations and wedge shaped infarcts), the well-recognized radiologic signs of pulmonary IMI are macronodules (≥1 cm in diameter), halo sign and air-crescent sign. Of these, macronodules are probably the most common. In one study, ~94% of patients with invasive pulmonary aspergillosis (IPA) presented with at least one macronodule on CT, suggesting that in high-risk patients, the absence of a macronodule argues against a diagnosis of IPA [20]. *Nodules* are characteristic of angioinvasion, a form of IPA that typically occurs in neutropenic or severely immunocompromised patients. Nodules may be surrounded by a ground glass opacity of non-inflammatory alveolar edema or hemorrhage called halo sign. *Halo sign* is the earliest CT chest manifestation of pulmonary IMI, often vanishing within a few days after onset of infection [21]. In severely immunocompromised hosts, the halo sign is highly suggestive of infection due to angioinvasive fungi, especially *Aspergillus* [22]. The true incidence of the halo sign among patients with HM and HSCT with IMI has varied widely, ranging from 25%–95% [21]. The *reversed halo sign* represents a focal rounded area of ground-glass opacity surrounded by a ring of consolidation and is most commonly associated with invasive pulmonary mucormycosis [22]. *Air-crescent sign*, a crescentic pocket of gas that is caused by retraction of necrotic lung from adjacent viable lung, typically appears later in the course of IMI, following neutrophil recovery [20,21]. IMI can also affect the airways leading to bronchiolar wall destruction, centrilobular micronodules and tree-in-bud opacities [23].

It is important to note that above described radiographic findings have been validated mostly in neutropenic patients with HM and HSCT recipients and are not as useful in other patient populations [24]. For example, the halo sign rarely occurs in immunocompromised but non-neutropenic/non-HM patients with IMI. Furthermore, none of these features are pathognomonic for IMI, as many other infections (such as tuberculosis, nocardiosis, other bacterial infections) and non-infectious conditions (such as granulomatosis with polyangiitis, post-transplant lymphoproliferative disorders) may also have similar CT manifestations [25,26].

In limited studies of SOT patients, the most common CT manifestations of IFI (mostly comprised of IPA) were: ground glass opacification, peribronchial consolidation, macronodules, and mass-like consolidations [24,27]; halo and air-crescent signs were uncommon. Indeed, in SOT recipients, it is the presence of pulmonary nodules and not the halo sign that should prompt investigation for and treatment of IMI [24,28].

HRCT pulmonary angiography (CTPA), by its ability to show vessel occlusion and/or decreased perfusion in pulmonary nodules, might improve the sensitivity and possibly the specificity of HRCT in diagnosing IMI among patients with HM [29,30]. This modality is based on the premise that angioinvasion with blood vessel occlusion is the hallmark of IMI; they are the earliest signs of IMI and precede the development of the halo sign. The sensitivity and specificity of CTPA for diagnosing proven or probable pulmonary IMI in small case series has ranged from 80%–100%, with a negative predictive value exceeding 90% [29]. CTPA may therefore be a good adjunct to the diagnosis of IMI in patients with HM and HSCT. However, more clinical data are needed to support the routine use of CTPA, since it carries a risk of contrast dye-induced nephropathy.

Positron Emission Tomography CT (PET/CT) using flourodeoxyglucose (FDG) as a biomarker has been used anecdotally in diagnosing and monitoring IFIs [31]. FDG accumulates in metabolically active cancer and inflammatory cells, and PET/CT has traditionally been used for staging and monitoring of malignancies. However, FDG is a nonspecific tracer and has been found to accumulate within migratory inflammatory cells and granulation tissue at the sites of infection [31,32]. It is believed that the increased FDG uptake is due to upregulation of cellular glucose metabolism in inflammatory cells [31,32]. FDG PET/CT has been reported to be positive in several IFIs (chronic mucocutaneous candidiasis, cryptococcosis, dimorphic and mould infections) [31,33]. In a retrospective study of 24 consecutive patients who underwent FDG PET/CT (eight with IPA and 16 with non-invasive aspergillosis), a hypermetabolic nodule pattern on FDG PET/CT was highly associated with IPA (occurring in 75% of patients with IPA), whereas an isometabolic halo pattern and an isometabolic nodule pattern on FDG PET/CT was highly associated with a non-invasive form of aspergillosis [34]. There is still limited experience regarding the role of FDG PET/CT in diagnosing IFIs, and further studies are needed. The ultimate role of this modality might be to evaluate the extent of disease and to monitor response to antifungal therapy.

## 4. Detection of Circulating Antigens

### 4.1. Galactomannan (GM) Detection

GM is a heteropolysaccharide cell wall component of *Aspergillus* that is released by germinating conidia and growing hyphae [5,35,36]. Most assays detect circulating GM in the blood and bronchoalveolar lavage fluid (BALF). A double-sandwich enzyme-linked immunosorbent assay (ELISA) (Platelia™ *Aspergillus* enzyme immunoassay, Bio-Rad Laboratories, Hercules, CA, USA) performed on serum and BALF has been approved by the US Food and Drug Administration (FDA) as an adjunct to the diagnosis of invasive aspergillosis (IA). The cut-off for positivity recommended by the manufacturer is an optical density index (ODI) of 0.5 for serum and BALF, and this is the value currently recommended by the FDA [37]. However, the best cut-off to define a positive result continues to spark debate [37,38,39].

#### 4.1.1. GM in Serum

In a meta-analysis of 27 studies, the pooled sensitivity and specificity of serum GM for patients with proven or probable IA were 71%–79% and 81%–86%, respectively (Table 1) [40,41]. However, these values showed marked variability across patient populations, study designs, and definitions of IA [37,38,41,42,43]. For instance, the sensitivity of serum GM in patients with HM and HSCT with IA was 70% and 82%, respectively, while the specificity was 92% and 86%, respectively [41]. Along the same lines, the sensitivity and specificity for SOT recipients with IA were 22% and 84%, respectively (Table 1) [41]. It is therefore prudent to keep in mind that the test fares differently across different hosts. Lastly, the performance of the test is negatively impacted by the concomitant use of anti-mould antifungals [38,43,44,45]. Thus, a “one size fits all” approach to testing may not apply. Mechanistically, this may be because neutropenic patients with HM and HSCT are not able to confine the disease to the lung [37]. They are thus more likely than SOT recipients to develop the angioinvasive form of the disease with penetration of hyphae through the endothelial cell layer, leading to release of the GM into the systemic circulation [36,38]. Similarly, those without neutropenia develop less extra-pulmonary dissemination, compromising the sensitivity of the serum GM assay [44].

The serum GM’s greatest utility lies in the serial screening of patients with HM and HSCT at high risk for IA [5,39]. These include patients with acute myelogenous leukemia undergoing intensive chemotherapy and patients receiving allogeneic HSCT during the early engraftment phase, in whom a diagnosis of IA by serum GM testing usually precedes the diagnosis by conventional diagnostic methods by a median of 8 days [5,39,46,47]. In these high-risk groups, serum GM had an excellent performance, with a high sensitivity and specificity of 92% and 98%, respectively, when two consecutive serum GM ODI were ≥0.5 [46]. Indeed, recent guidelines from the European Conference on Infections in Leukemia (ECIL) recommend GM monitoring every 3–4 days in this patient population, while incorporating HRCT imaging and appropriate clinical and microbiological evaluation as indicated [39].

**Table 1:** Diagnostic performance of GM, BDG and A*spergillus* PCR and recommendations for their roles in diagnosis and screening. Unless indicated, performance data are derived from meta-analysis. ^¥^ Limited data, data shown based on individual reports.

Assays	Sensitivity	Specificity	Recommendations	Caveats
**Serum GM**				
All studies [40,41]	71%–79%	81%–86%	-	Sensitivity is impacted by anti-mould antifungals; Many causes of false positive tests.
HSCT [41]	82%	86%	Diagnosis of IA: moderate performance. Can be used as adjunct to other diagnosis modalities; Screening for IA (2 to 3 times a week), in adjunct with serum or blood PCR. Results can be used to trigger biomarker-driven antifungal therapy.	
SOT [41]	22%	84%	Diagnosis of IA: poor sensitivity. Can be used as adjunct to other diagnosis modalities.	
**BALF GM**				
All studies [38,43]			Diagnosis of IA: good at cut-off of 1.0. Negative BALF GM essentially rules out IPA if the patients are not on anti-mould antifungals.	-
Cut-off ODI of 0.5 Cut-off ODI of 1.0 Cut-off ODI of 1.5	86%–87% 85%–86% 70%–85%	89% 89%–95% 95%–96%		
Hem malignancies/HSCT [48]			Diagnosis of IA: good at cut-off of 1.5. Negative BALF GM essentially rules out IPA if the patients are not on anti-mould antifungals.	Optimal cut-off for positivity not clear (probably 1.0 or 1.5); Sensitivity might be impacted by anti-mould antifungals.
Cut-off ODI of 0.5 Cut-off ODI of 1.0 Cut-off ODI of 1.5	82% 72% 92%	92% 95% 98%		
**SOT ^¥^**				
Organ transplant [49,50]			Diagnosis of IA: good. Negative BALF GM essentially rules out IPA if the patient is not on anti-mould antifungals; GM should not be routinely tested in surveillance BALF in lung transplant patients due to low specificity.	Optimal cut-off for positivity not clear (probably 1.0 or 1.5) GM in BAL cannot differentiate IPA from *Aspergillus* colonization.
Cut-off ODI of 0.5 Cut-off ODI of 1.0 Cut-off ODI of 1.5	82%–100%82%–100% 100%	84%–96%91%–97% 92%		
Lung transplant [51,52]				
Cut-off ODI of 0.5 Cut-off ODI of 1.0 Cut-off ODI of 1.5	60–100% 60–100% 100%	40%–95% 81%–98% 90%		
**Serum BDG**				
All studies [53] (all IFI) All studies [53] (IA only)	77% 77%	85% 83%	-	Panfungal diagnostic test thus cannot differentiate between fungal pathogens; Many causes of false positive tests; Sensitivity is impacted by antifungals.
Hem malignancies/HSCT [39]			Diagnosis of IFI: Utility of the test is hindered by low specificity. Cannot identify specific fungal pathogen responsible for infection; Screening for IFI: mixed recommendations by experts. Low accuracy has been reported among patients with hematologic malignancies. Many centers prefer serum GM over BDG for screening or monitoring purpose; Performance may be increased with serial testing (2 consecutive positive results).	
Single positive test	77%	85%		
Two consecutive positive tests	50%	99%		
SOT ^¥^	66%	44%	Very limited data. Not useful in lung transplant patients because of very low PPV.	
**Serum/Blood PCR**				
All [54,55]	84%–88%	75%–76%	-	Wide range of diagnostic performance due to non-standardized methodology and study design.
≥1 positive test ≥2 positive tests	64% 64%	64% 95%		
Hem malignancies/HSCT [39]	88%	75%	Diagnosis of IA: strongly consider IA with 2 consecutive positive tests; Screening for IA (2 to 3 times a week), may be done in adjunct with serum GM. Results can be used to trigger biomarker-driven antifungal therapy.	
SOT ^¥^	No data	No data	No data	
**BALF Aspergillus PCR**				
All [42], [56]	90%–91%	92%–96%	-	Non-standardized methodology; Many causes of false positive tests
Hem malignancies/HSCT [48]	57%	99%	Diagnosis of IPA: fair to good (the sensitivity from the meta-analysis was lower than previously published rates).	The corresponding performances of GM with BALF were 79% and 97%, respectively.
SOT	100%	88%	Diagnosis of IPA: good to very good. Cannot differentiate between IPA and fungal colonization.	-

Serum GM has also been evaluated for guiding targeted antifungal therapy for a subset of high-risk neutropenic patients [57]. This approach, which incorporated serial serum GM screening and chest CT to detect IA among neutropenic patients with leukemia who received fluconazole prophylaxis, reduced empiric anti-mould antifungal therapy by 78%, and led to the early initiation of antifungal therapy in 7% of episodes that were clinically not suspected to be IFIs [57].

Since the GM ODI correlates with disease burden, GM monitoring should be continued after antifungal therapy has been initiated [39]. Very high GM antigenemia at the beginning of treatment portends a worse outcome, and persistent GM antigenemia during therapy is a poor prognostic sign [39,45]. Unfortunately, these early screening strategies have not been successfully reproduced in other IA at-risk groups, including SOT recipients.

#### 4.1.2. GM in BALF

Testing GM in BALF fluid yielded superior sensitivity (86%–87%) but lower specificity (89%) than serum for the diagnosis of IPA when the GM ODI threshold of 0.5 was used (Table 1) [40,41,43,48]. The superior sensitivity is likely due to the higher fungal burden in the lungs of patients with IPA [43]. However, a lower specificity was observed in BALF fluid of lung transplant recipients whose airways are often colonized with *Aspergillus* and other moulds, thereby limiting the usefulness of BALF GM testing in surveillance bronchoscopy [37,49,51,52]. BALF testing has also performed well in other non-neutropenic populations such as those with SOT [49,50,52,58], chronic obstructive pulmonary disease (COPD) or mechanical ventilation [37]. The manufacturer recommends a similar BALF GM ODI cut-off of 0.5 (as in serum), but a cut-off of 1.0 or 1.5 might be more appropriate. Indeed, when the GM index threshold is raised to these values, the specificity of the test improves without sacrificing its sensitivity [38,43].

Lastly, a meta-analysis demonstrated that, among patients with HM, a combination of serum and BALF GM at a cut-off of 0.5 improved sensitivity to 90% (from 78% and 81% with serum and BALF, respectively) and reduced specificity to 89% (from 98% and 93% with serum and BALF, respectively).

#### 4.1.3. GM at Other Sites

GM has been detected in sputum, urine, cerebrospinal fluid (CSF), pleural fluid and homogenized tissue specimens among patients with IA [5,59]. However, the experience with GM detection outside of blood and BALF is very limited, and thus the diagnostic yields are not known.

#### 4.1.4. Limitations

Several limitations of GM testing have been noted. First, the turnaround time varies between centers (between less than a day to several days), and specimens often need to be sent to reference laboratories for processing [36,60,61,62]. Results may therefore be available too late to be clinically useful. Second, various causes of false-positivity have been described (Table 2). Of importance, false positive GM has been classically described in the setting of concurrent use of piperacillin-tazobactam, a semi-synthetic penicillin that is produced by *Penicillium*, which, like *Aspergillus*, contains GM in its cell wall [37,63]. However, current formulations of piperacillin/tazobactam no longer contain residual GM, making it unlikely that administration of this drug will result in a false-positive GM result [63]. Finally, as mentioned previously, the sensitivity of GM is reduced in patients receiving mould-active antifungal agents [38,43,44,45]. Thus, GM is not recommended for screening of asymptomatic patients receiving mould-active antifungal agent.

**Table 2:** Common causes of false positive GM, BDG and PCR tests.

GM	BDG	PCR
Semisynthetic antibiotics based on natural compounds derived from the genus *Penicillium* such as piperacillin, amoxicillin-clavulanate	Hemodialysis with cellulose membranes	Contaminated blood/serum/urine collection tubes, PCR tubes, PCR reagents
Colonization or infection due to other fungi: *Penicillium*, *Fusarium*, *Paecilomyces*, *Histoplasma*, *Blastomyces*	Receipt of IV immunoglobulin, albumin, or other blood products filtered through cellulose depth filters containing BDG	Colonization with *Aspergillus* spp., including environmental non-pathogenic *Aspergillus* spp.
Receipt of blood transfusion or other blood-derived products. Utilization of Plasmalyte for BAL	Gauze packing of serosal surfaces	Colonization or infection due to other fungi: *Penicillium*
Food products (pasta, rice, *etc*)	Bacterial bloodstream infections, such as *Pseudomonas aeruginosa*	Environmental fungal contamination

### 4.2. 1,3-β-d Glucan (BDG) Detection

BDG is a pan-fungal diagnostic assay that detects a non-antigenic cell wall component of most pathogenic fungi, including *Aspergillus*, *Candida* and *Pneumocystis*, but not *Mucorales* and *Cryptococcus*. Four assays are currently commercially available and differ in their methods of detection (colorimetric *versus* turbimetric) and the substrates used for the chromogenic reaction. Consequently, there are differences in the optimal thresholds for test positivity. In the US, the Fungitell assay (Associates of Cape Cod, East Falmouth, MA, USA) has been approved by the FDA for the presumptive diagnosis of IFI. Given the non-specific nature of the test, it should be used in conjunction with other diagnostic markers. The manufacturer recommends the cut-off for positive and negative tests be ≥80 pg/mL and <60 pg/mL, respectively. Levels of 60–79 pg/mL are considered indeterminate. However, better performance of the test has been reported with higher cut-off values.

#### 4.2.1. BDG in Serum

In most studies, BDG testing was used to detect all types of IFIs. In a meta-analysis, the pooled sensitivity and specificity for BDG among patients with IFI were 77% and 85%, respectively (Table 1) [53]. As with GM, these data should be interpreted with caution, as the analysis was afflicted by the heterogeneity in study designs, characteristics of the study population, types of pathogens evaluated, assays and cut-off levels used, sampling methods, definitions of positive test results, and prior administration of antifungal therapy [53]. Indeed, another meta-analysis focusing solely on patients with HM revealed a lower sensitivity (70%) and better specificity (91%) [37]. Due to its low sensitivity, a negative test cannot rule out IFI. There is limited information on the diagnostic performance of BDG specifically for IA, but a subgroup analysis of 17 studies with IA revealed similar performance characteristics for IC and IA (sensitivity of 77% and specificity of 83%, respectively, for IA).

Results of direct comparative studies between BDG and GM are conflicting, with some studies favoring BDG, some favoring GM, while others found similar performances [60,64,65,66]. One study suggested that BDG was detected ≥4 days earlier than GM in ~30% of patients [67]. Overall, the limitations of BDG are its inability to distinguish infections due to various fungal species (*Candida*, *Saccharomyces*, *Trichosporon*, *Coccidioides*, *Histoplasma*, *Sporothrix*, *Aspergillus*, *Fusarium*, *Acremonium*, *Pneumocystis*, *etc.*) and its low specificity (Table 2).

There have been emerging data that inclusion of two consecutive positive BDG tests in the diagnostic criteria improves the specificity (up to 99%), without a significant effect on the sensitivity (remains low ~50%) [53]. Along the same lines, combining BG and GM assays improves the diagnosis of IA by eliminating the false positivity of either individual test alone [64].

To date, the BDG has generally been evaluated for patients with HM and those hospitalized in intensive care units. Unlike the experience with screening patients with HM and HSCT for IA during the highest-risk period using serial GMs two or three times per week, BDG screening has limited value among patients with HM due to its low sensitivity and extremely low positive predictive value (10%–12%) [68].

Only a few studies have assessed the utility of BDG among SOT recipients, in whom the sensitivity and specificity of the test were 66% and 44%, respectively (Table 1) [69]. Particularly, the performance of BDG among lung transplant patients is marginal [70]. For example, 90% of lung transplant patients with positive BGD did not have IFI; this low positive predictive value limits its use in screening for IFI.

BDG also detects *Pneumocystis* *jirovecii* pneumonia (PCP). A meta-analysis demonstrated that BDG had excellent sensitivity in detecting PCP among patients with the acquired immunodeficiency syndrome (AIDS), HM, and others [71]. The sensitivity of BDG in detecting PCP was 95%, much higher than sensitivity in detecting IA and IC, but the specificity was similar for all three infections [53,71]. In addition, several studies have found very high BDG levels (Fungitell assay with levels >500 pg/mL) among patients with PCP [71]. It has been recently recognized that *P.* *jirovecii* can colonize the respiratory tracts of patients with chronic pulmonary diseases and immunocompromised states [72], and colonization causes false positive BDG in serum and BALF. However, levels of BDG associated with PCP have been shown to be higher than in *P.* *jirovecii* colonization [73,74]. These data altogether suggest that the current optimal cut-off for BDG test positivity in PCP is not known, and as with other diagnostic tests, the results of BDG should be interpreted in conjunction with symptomatology and radiologic findings.

The utility of BDG in other less common IFI is not known due to lack of data. Few studies have documented positive BDG test among patients infected with fusariosis and phaeohyphomycosis [27].

#### 4.2.2. BDG Detection at Extra-Blood Sites

In general, BDG is not recommended for routine testing of BALF because this is commonly colonized with *Candida* spp. [75]. In a study of 132 BALF samples from patients with pneumonia (among whom 10 had PCP, 14 had IPA, and 20 had other IFIs), BALF BDG had comparable sensitivity to BALF GM in detecting all IFIs (53% *versus* 42%); this was a disappointing result, since BDG did not improve detection of non-Aspergillus moulds that cannot be detected by GM [76]. The sensitivities of BALF BDG for detecting PCP and IPA were 100% and 71%, respectively; this was similar to the sensitivity of BALF GM for diagnosing IPA. As expected, BDG testing exhibited poor specificity (38%–68%) in BALF [76]. Of importance, poor reproducibility of the test is noted in BALF: upon repeat testing, only 6% of BALF BDG samples yielded consistent results, compared to 77.3% of serum BDG samples.

BDG has also been detected in the CSF of patients with meningitis. The largest study involved CSF from 41 patients with either PCR- or culture-confirmed cases of meningitis due to *Exserohilum restratum* (patients involved in a contaminated methylprednisolone outbreak) and 66 patients without meningitis [77]. A cut-off value of 138 pg/mL resulted in excellent performance with 100% sensitivity and 98% specificity for the diagnosis of fungal meningitis in this contaminated methylprednisolone outbreak [77]. Furthermore, among patients with serially-collected CSF, BDG levels appeared to correlate with clinical response. BDG has also been detected in CSF for a wide range of IFIs including *Aspergillus*, *Candida*, *Histoplasma*, *Fusarium* and other non-*Aspergillus* moulds [78,79].

## 5. Molecular Approaches

Although polymerase chain reaction (PCR) for the diagnosis of IA has been reported for over two decades, it has not been widely used in the US because of a lack of standardization of the assay. A recent meta-analysis of PCR in blood has noted heterogeneity in the nature of samples studied (whole blood, serum, plasma), volume of sample tested, specimen processing and DNA extraction methods, gene targets, amplification platforms, detection methodologies, and definitions of PCR positivity [54,55]. In addition, study design, target patient population, and the inclusion of one or >1 PCR result (which has been shown to improve specificity) to define positivity also vary between studies. This has resulted in variable performances, with the sensitivities ranging from 36%–100%, and specificities from 80%–96%.

Overall, the pooled sensitivity (84% among studies with ≥1 positive results and 64% among studies with ≥2 positive results) and specificity of blood or serum PCR (respective performance of 64% and 95%) [55] were not inferior to those of serum GM (71 and 89%, respectively) and BDG (77% and 85%, respectively) obtained from previous meta-analyses [41,53]. It is clear from a recent meta-analysis of *Aspergillus* PCR from blood or serum that a single positive or negative PCR result can neither confirm nor exclude IA. However, when two positive PCR results were used to define a positive test, the specificity and positive predictive value of the assay improved to 95% and 90%, respectively [55]. This suggests that the diagnosis of IA should be seriously considered in high-risk patients with two consecutive positive PCR results.

*Aspergillus* PCR in BALF performs better than in blood. In a systematic review of nine studies using reference IA definitions strictly adherent to the EORTC/MSG criteria, the sensitivity and specificity of PCR of BALF were 77% and 94%, respectively [42]. As with the meta-analyses of serum PCR, this analysis was also subjected to heterogeneity regarding DNA extraction, primer designs and PCR methodology. Overall, DNA extraction from BALF cell pellets and utilization of commercial kits for cell wall disruption and DNA extraction yielded better sensitivity [56,80]. It is important to note that *Aspergillus* PCR in BALF cannot differentiate colonization from invasive infection. However, the high negative predictive value of BALF PCR (usually ≥95%) suggests its role in ruling out IPA. To date, the diagnostic performance of blood and BALF PCR appears to be comparable to that of serum and BALF GM (cut-off index of ≥0.5), respectively, and the sensitivity for both tests is affected by anti-mould antifungal use. Using either GM or PCR to define test positivity in BALF resulted in improved sensitivity (97%) with no sacrifice of specificity (98%) [42].

Small studies of *Aspergillus* PCR on non-blood and extra-pulmonary body fluid samples (CSF, various effusion fluids) and paraffin-preserved and fresh tissues (lung, skin, sinus, lymph node) demonstrated sensitivity of 86% and specificity of 100% [81,82,83]. However, the sensitivity of PCR in pleural fluid was much lower than in fresh tissues, in the 40% range [82].

Factors affecting the performance of A*spergillus* PCR are multi-fold. From earlier studies, whole blood was favored over serum due to better sensitivity [84,85]. One study showed a trend for whole blood to be more sensitive (85% *versus* 79%) and to yield an earlier positive result (36 days *versus* 15 days) than for serum [85]. However, subsequent studies and a recent meta-analysis failed to show a difference in performance between the two blood compartments [55,85,86]. PCR using whole blood, however, suffers from false positivity and low specificity compared with serum specimens. Since serum is also easier and faster to process than whole blood and can be used for simultaneous GM testing [85], serum might potentially become the preferred choice for blood PCR. In general, plasma is not suitable for PCR testing since it contains anticoagulants that might inhibit the enzyme used in the amplification process. Indeed, for IA diagnosis, the sensitivity of PCR performed in plasma was much lower than that performed in whole blood or serum [85].

DNA extraction from samples and PCR methodologies may also affect PCR test performance. In this regard, great effort has been devoted to the standardization of the *Aspergillus* PCR assay. First, initiatives such as the European *Aspergillus* PCR Initiative (EAPCRI) have developed standards for *Aspergillus* blood-based PCR methodologies and a calibrator in collaboration with the *Aspergillus* Technology Consortium [68,87,88,89,90,91]. It identified that the efficiency of the *Aspergillus* PCR is limited by the DNA extraction procedure, rather than PCR amplification [85,88]. Furthermore, greater sensitivity can be achieved with the use of larger sample volumes (≥3 mL), an internal control PCR, and PCR targeting the internal transcribed spacer (ITS) region [88]. Lower sensitivity was linked with larger elution volumes (≥100 μL) and PCR targeting the mitochondrial genes [88]. In a meta-analysis, compliance with the EAPCRI recommendations significantly improved performance [55].

Second, centralization of PCR testing at a reference laboratory is another means for standardizing the assay. For example, a genus- and species-specific quantitative *Aspergillus* PCR, comprised of three real-time PCR assays that detect all *Aspergillus* species, *A. fumigatus* and *A. terreus* within the respiratory specimens is currently available as a fee-for-service (ViraCor-IBT Laboratories, Lee’s Summit, MO, USA). Using a rabbit model of IPA due to *A. fumigatus*, the sensitivities of pan-*Aspergillus* PCR and *A. fumigatus*-specific PCR in BALF were higher than those of culture (100% and 96%, respectively, *vs.* 50%), but comparable to GM (92%) [92]. When this assay was tested on 150 BALF samples from lung transplant recipients, the sensitivity and specificity of pan-*Aspergillus* PCR for diagnosing IPA were 100% and 88%, respectively, comparable to those of GM at a cut-off of ≥0.5 (93% and 89%, respectively) [51]. The sensitivity and specificity of *A. fumigatus*-specific PCR were 85% and 96%, respectively. *A. terreus*-specific PCR was positive for the one patient with IPA due to this species. It was clear from this study that PCR cannot differentiate IPA from *Aspergillus* colonization. Indeed, for BALF associated with *Aspergillus* colonization, the specificity of GM (92%) was higher than that of pan-*Aspergillus* PCR (50%). Among negative control samples, the specificity of pan-*Aspergillus* PCR (97%) was higher than that of BALF GM (88%). Positive results of both BALF PCR and GM testing improved the specificity to 97% with minimal detriment to sensitivity (93%). Unfortunately, the experience with this PCR assay has not been validated in other populations at risk for IPA, such as patients with HM or HSCT recipients.

Third, commercialization of the assay also provides a means to standardization of the methodology and quality control of the reagents. Several PCR assays are currently commercially available outside the U.S. The most well-studied assay to date is the MycAssay^®^ *Aspergillus* (Myconostica, Cambridge, UK). This is a real-time PCR assay targeted at 18S ribosomal RNA for the detection of *Aspergillus* DNA in serum and lower respiratory tract samples. Small scale studies in serum revealed sensitivity of 44% to 47% and specificity 63–98% for diagnosis of IA [93,94,95]. Overall, it has a high concordance with the serum GM assay. Several studies have shown similar performance to GM in serum [93,96], whereas one study of patients with HM showed lower specificity than GM [94]. Better performance for this assay was achieved with testing BALF samples: small scale studies have shown sensitivities of 80%–100% and specificities of 92–97% [97,98]. It is important to point out that the MycAssay^®^ *Aspergillus* kit also amplifies Penicillium spp. as *Aspergillus* and *Penicillium* have identical 18S target sequences, and false positivites have been documented in BALF growing *Penicillium.*

AsperGenius (Pathonostics, Maastricht, The Netherlands) is a commercially developed multiplex real-time PCR *Aspergillus* detection assay that has the added ability to detect azole resistance in *A. fumigatus.* The limits of detection of the species and resistance assays when detecting *A. fumigatus* DNA were 10 and ≥75 genomes/sample, respectively. The assay was validated for testing in serum of 14 cases of proven/probable IA, two cases of possible IA and 33 controls, where the sensitivity and specificity were 79% and 100%, respectively [99]. The assay was also validated using 37 and 40 BALF samples from patients with HM and those residing in intensive care units, respectively (11 proven/probable IA and 11 non-classifiable) [100]. In this cohort, the sensitivity and specificity were 89% and 89%, respectively. This assay also detected azole mutations directly from eight BALF associated with IA.

Overall, although commercial assays enable the standardization of PCR, several potential limitations should be noted. First, most assays have a pan-*Aspergillus* target, thus detecting both pathogenic and non-pathogenic environmental *Aspergillus* spp. Second, PCR detects fungal DNA which can originate from either live or dead conidia or hyphae, and thus might not be ideal for monitoring the efficacy of antifungal therapy. Third, since the target for detection is broad, the assays have cross-reactivity to other moulds, including *Penicillium*. Last but not least, commercially available products such as blood/serum/urine collection tubes, PCR plastic tubes *etc.* have been shown to be contaminated with *Aspergillus* DNA [101]. Since real-time PCR is able to detect very low concentrations of *Aspergillus*, these issues altogether suggest that PCR assays might be challenged with false positivity.

PCR has also been developed for the diagnosis of mucormycosis. In addition to the inherent problems experienced with *Aspergillus* PCR, the design of mucormycosis PCR is further complicated by the wide range of species involved and the low incidence of disease. Nevertheless, a quantitative multiplex PCR consisting of hydrolysis probes targeting the common mucormycosis geni (*Mucor/Rhizopus*, *Lichtheimia*, and *Rhizomucor*) has been developed and tested in a series of ten patients with proven mucormycosis and yielded promising results [102]. Importantly, the serum PCR was positive in 90% of patients between 68 and three days before the diagnosis was made by histopathology and/or culture. All PCR results were concordant with culture and/or PCR-based identification of the causative pathogens in tissues [102,103]. Other locally-made PCR products also yielded promising detection capabilities of mucormycosis using serum, BALF, and tissues [9].

Panfungal PCR has been developed to detect a broad range of IFIs in tissue samples and blood. However, this generally requires follow-up genus-specific PCRs or DNA sequencing of the amplicon for identification of the specific fungal pathogen. Limited studies to date have shown that panfungal PCR is more sensitive than culture or histology and is able to identify a wide spectrum of fungi [83,104,105]. It might have a promising role as adjunct to culture and histopathology.

## 6. Lateral Flow Device

The lateral flow device (LFD) is a novel “point-of-care” immunochromatographic assay that uses a monoclonal JF5 antibody to detect a glycoprotein antigen secreted by *Aspergillus* species during active growth [36]. It is easily performed, yields results within 15 minutes, and can be done in any laboratory [36,60,62,106]. The few studies that have investigated its performance on BALF have shown promising results, comparable but not superior to those of GM or PCR testing. In a study of patients with underlying respiratory disease but without neutropenia or transplantation, it had a sensitivity of 77% and specificity of 85%–92% [36]. In contrast, another study in SOT recipients demonstrated a sensitivity and specificity of 91% and 83%, respectively, for patients with proven or probable IA [60]. Its performance in serum samples has varied however, with a sensitivity ranging from 40%–81% and a specificity of 85% [61,106]. The lower sensitivity in one study may have been because BDG positivity was used as a criterion to define invasive mould disease, thus incorporating non-*Aspergillus* fungi that would not be detected by LFD [61].

While in theory LFD should only be able to detect *Aspergillus*, in practice false positive results have been reported in patients with *Penicillium* and *Candida* in their respiratory tract [60,62]. Further, it may not always reliably differentiate colonization from invasive disease [60]. However, it can remain positive even after the GM has been rendered negative by prior antifungal therapy [62]. Another potential problem is that interpretation of LFD results is subjective and relies on operator interpretation of test line intensities that can range from negative to strongly or weakly positive [62,106]. One potential solution for this includes implementing an “all or none” approach for interpretation [106]. Alternatively, handheld densitometers can be used to measure the color intensity of the test line and allow establishment of detection thresholds [106].

Thus, while not without its shortcomings, the rapid turnaround time of the LFD makes it an attractive option for the timely diagnosis of IA, either alone or combined with other assays [106]. Larger studies are needed before implementation into routine clinical practice can be recommended.

## 7. Breath Test

Exhaled air contains volatile organic compounds (VOCs) derived from various metabolic pathways. These VOCs have been used as biomarkers for infectious diseases [107] and were recently tested as a potential diagnostic tool for IA. Two reports documented the presence of 2-pentyfuran in the breath of patients colonized or infected with *Aspergillus* [108,109]. They were subsequently followed by a proof-of-concept study evaluating an electronic nose as a mechanism to detect VOC signatures of *Aspergillus* in a small cohort of patients with chemotherapy-induced prolonged neutropenia (six IA and five controls). The sensitivity and specificity were 100% and 83%, respectively [110]. A more sophisticated study using thermal desorption-gas chromatography/mass spectrometry of 34 patients with IA and 30 controls demonstrated that the presence of specific VOCs in the breath of patients was able to discriminate between patients with proven/probable IA from those with other IFI or other causes of pneumonias, with a sensitivity and specificity of 94% and 93%, respectively [111]. These findings are promising but need to be validated in larger scale studies before breath testing can be widely applied.

## 8. Application of Biomarkers

Empiric antifungal therapy has been traditionally administered to patients at risk for IFI, such as in the setting of persistent neutropenic fever despite broad-spectrum antibacterial therapy. However, this approach is costly, with the potential for over-treatment leading to drug-related toxicities and increased cost; there is also a theoretical concern about the emergence of antifungal resistance. The introduction of non-invasive and sensitive markers to detect IA has led to the conduction of several trials of biomarker-driven antifungal therapy among patients with HM at high-risk for IA. In a recent trial comparing biomarker-based diagnostic screening (twice weekly GM and *Aspergillus* PCR) with a standard diagnostic strategy (histopathology and culture) among leukemic patients receiving chemotherapy or HSCT recipients, the use of biomarkers reduced empiric antifungal treatment, identified more cases of IA, and was not associated with worse outcomes [112]. In this trial, no cases of IA were missed. Economic evaluation of the same study showed that total costs were 32% lower for the biomarker-driven strategy due to a reduced incidence of adverse events and decreased antifungal usage [113]. A subsequent study evaluated the impact of serum PCR when this test was used in conjunction with serum GM on the early diagnosis and therapy of IA using similar high-risk patients [114]. By adding serum PCR to GM monitoring, the time from the start of monitoring to diagnosis was shortened by a median of 7 days, and the need for empiric antifungal therapy was also reduced. More importantly, combined GM-PCR screening significantly decreased the incidence of IA. Although the simultaneous use of GM and PCR enhanced the identification of patients at risk for IA, a potential limitation of this approach is the higher cost and requirement of specialized laboratories for test performance. Nevertheless, these studies and others suggest that the biomarker-driven strategies are associated with less unnecessary antifungal use without compromising overall survival, and this can be safely applied to clinical practice. It is important to point out that screening and biomarker-driven antifungal usage has been only studied in patients with HM and HSCT in the high-risk period for IA, in whom antifungal prophylaxis consisted of either fluconazole or itraconazole. These screening strategies have also not been evaluated for SOT recipients. Therefore, this practice cannot be applied to patients with HM or HSCT receiving voriconazole or posaconazole for prophylaxis, or patients undergoing SOT.

## 9. Conclusions

Although significant progress has been made in developing non-culture diagnostics, to date there is not one specific test that can accurately diagnose IA or IFI. Therefore, diagnosis relies on complementary approaches that incorporate clinical criteria, radiographic findings, histopathology, culture and new non-culture diagnostics. Work in progress includes the standardization of molecular methodologies to assure intra- and inter-laboratory reproducibility, optimization of PCR performance, and clinical validation of the molecular assays. In the future, the hope is to utilize non-culture diagnostics to both improve the identification of IA/IFI and shorten the time to diagnosis. In addition, a goal is to develop management strategies that incorporate non-culture diagnostics and facilitate more timely antifungal therapy among patients with HM and HSCT. The feasibility and costs of performing tests in the clinical laboratory will need to be assessed.
